# SARS-CoV-2 Breakthrough Infections: Incidence and Risk Factors in a Large European Multicentric Cohort of Health Workers

**DOI:** 10.3390/vaccines10081193

**Published:** 2022-07-27

**Authors:** Stefano Porru, Maria Grazia Lourdes Monaco, Gianluca Spiteri, Angela Carta, Maria Diletta Pezzani, Giuseppe Lippi, Davide Gibellini, Evelina Tacconelli, Ilaria Dalla Vecchia, Emma Sala, Emanuele Sansone, Giuseppe De Palma, Carlo Bonfanti, Massimo Lombardo, Luigina Terlenghi, Enrico Pira, Ihab Mansour, Maurizio Coggiola, Catalina Ciocan, Alessandro Godono, Adonina Tardon, Marta-Maria Rodriguez-Suarez, Guillermo Fernandez-Tardon, Francisco-Jose Jimeno-Demuth, Rafael-Vicente Castro-Delgado, Tania Iglesias Cabo, Maria Luisa Scapellato, Filippo Liviero, Angelo Moretto, Paola Mason, Sofia Pavanello, Anna Volpin, Luigi Vimercati, Silvio Tafuri, Luigi De Maria, Stefania Sponselli, Pasquale Stefanizzi, Antonio Caputi, Fabriziomaria Gobba, Alberto Modenese, Loretta Casolari, Denise Garavini, Cristiana D’Elia, Stefania Mariani, Francesca Larese Filon, Luca Cegolon, Corrado Negro, Federico Ronchese, Francesca Rui, Paola De Michieli, Nicola Murgia, Marco Dell’Omo, Giacomo Muzi, Tiziana Fiordi, Angela Gambelunghe, Ilenia Folletti, Dana Mates, Violeta Claudia Calota, Andra Neamtu, Ovidiu Perseca, Catalin Alexandru Staicu, Angelica Voinoiu, Eleonóra Fabiánová, Jana Bérešová, Zora Kľocová Adamčáková, Roman Nedela, Anna Lesňáková, Jana Holčíková, Paolo Boffetta, Mahsa Abedini, Giorgia Ditano, Shuffield Seyram Asafo, Giovanni Visci, Francesco Saverio Violante, Carlotta Zunarelli, Giuseppe Verlato

**Affiliations:** 1 Section of Occupational Medicine, Department of Diagnostics and Public Health, University of Verona, 37134 Verona, Italy; angela.carta@univr.it; 2 Occupational Medicine Unit, University Hospital of Verona, 37134 Verona, Italy; mariagrazialourdes.monaco@aovr.veneto.it (M.G.L.M.); gianluca.spiteri@aovr.veneto.it (G.S.); 3 Infectious Diseases Unit, University Hospital of Verona, 37134 Verona, Italy; mariadiletta.pezzani@aovr.veneto.it (M.D.P.); evelina.tacconelli@univr.it (E.T.); 4 Section of Clinical Biochemistry, Department of Diagnostics and Public Health, University of Verona, 37134 Verona, Italy; giuseppe.lippi@univr.it; 5 Section of Microbiology, Department of Diagnostics and Public Health, University of Verona, 37134 Verona, Italy; davide.gibellini@univr.it; 6 Section of Infectious Diseases, Department of Diagnostics and Public Health, University of Verona, 37134 Verona, Italy; ilaria.dallavecchia@studenti.univr.it; 7 Unit of Occupational Health, Hygiene, Toxicology and Prevention, University Hospital ASST Spedali Civili, 25121 Brescia, Italy; emma.sala@unibs.it (E.S.); giuseppe.depalma@unibs.it (G.D.P.); 8 Unit of Occupational Health and Industrial Hygiene, Department of Medical Surgical Specialties, Radiological Sciences and Public Health, University of Brescia, 25121 Brescia, Italy; e.sansone@unibs.it; 9 Department of Molecular and Translational Medicine, Institute of Microbiology, University of Brescia-ASST Spedali Civili, 25121 Brescia, Italy; carlo.bonfanti@unibs.it (C.B.); luigina.terlenghi@asst-spedalicivili.it (L.T.); 10 Chief Executive Office, ASST Spedali Civili di Brescia, 25121 Brescia, Italy; direttore.generale@asst-spedalicivili.it; 11 Department of Public Health and Pediatrics, University of Turin, 10126 Turin, Italy; enrico.pira@unito.it (E.P.); ihab.mansour@unito.it (I.M.); catalina.ciocan@unito.it (C.C.); alessandro.godono@unito.it (A.G.); 12 Occupational Medicine Unit, University Hospital Città Della Salute e Della Scienza di Torino, 10126 Turin, Italy; maurizio.coggiola@unito.it; 13 Health Research Institute of Asturias (ISPA), CIBERESP and Public Health Department of the University of Oviedo, Campus del Cristo s/n, 33006 Oviedo, Spain; atardon@uniovi.es (A.T.); martamaria.rodriguezs@sespa.es (M.-M.R.-S.); gfernanta@gmail.com (G.F.-T.); jimenofrancisco@uniovi.es (F.-J.J.-D.); castrorafael@uniovi.es (R.-V.C.-D.); iglesiasctania@uniovi.es (T.I.C.); 14 Department of Cardiac Thoracic Vascular Sciences and Public Health, University of Padova, 35128 Padova, Italy; marialuisa.scapellato@unipd.it (M.L.S.); filippo.liviero@unipd.it (F.L.); angelo.moretto@unipd.it (A.M.); paola.mason.1@unipd.it (P.M.); sofia.pavanello@unipd.it (S.P.); 15 University Hospital of Padova, 35128 Padova, Italy; anna.volpin@aopd.veneto.it; 16 Interdisciplinary Department of Medicine, University of Bari, 70124 Bari, Italy; luigi.vimercati@uniba.it (L.V.); silvio.tafuri@uniba.it (S.T.); luigi.demaria@uniba.it (L.D.M.); stefania.sponselli@uniba.it (S.S.); pasquale.stefanizzi@uniba.it (P.S.); antonio.caputi@uniba.it (A.C.); 17 Department of Biomedical, Metabolic and Neural Sciences, University of Modena & Reggio Emilia, 41125 Modena, Italy; fabriziomaria.gobba@unimore.it (F.G.); alberto.modenese@unimore.it (A.M.); 18 Health Surveillance Service, University Hospital of Modena, 41125 Modena, Italy; casolari.loretta@aou.mo.it (L.C.); garavini.denise@aou.mo.it (D.G.); delia.cristiana@aou.mo.it (C.D.); mariani.stefania@aou.mo.it (S.M.); 19 Unit of Occupational Medicine, Department of Medical Science, University of Trieste, 34149 Trieste, Italy; larese@units.it (F.L.F.); or l.cegolon@gmail.com (L.C.); negro@units.it (C.N.); ronchese@units.it (F.R.); rui@units.it (F.R.); michieli@units.it (P.D.M.); 20 Section of Occupational Medicine, Respiratory Diseases and Toxicology, Department of Medicine and Surgery, University of Perugia, 06123 Perugia, Italy; nicola.murgia@unipg.it (N.M.); marco.dellomo@unipg.it (M.D.); giacomo.muzi@unipg.it (G.M.); tiziana.fiordi@ospedale.perugia.it (T.F.); angela.gambelunghe@unipg.it (A.G.); ilenia.folletti@unipg.it (I.F.); 21 National Institute of Public Health, 050463 Bucharest, Romania; dana.mates@insp.gov.ro (D.M.); violeta.calota@insp.gov.ro (V.C.C.); andra.neamtu@inps.gov.ro (A.N.); ovidiu.perseca@insp.gov.ro (O.P.); catalin.staicu@insp.gov.ro (C.A.S.); angelica.voinoiu@insp.gov.ro (A.V.); 22 Occupational Health Department, Regional Authority of Public Health, 97556 Banská Bystrica, Slovakia; fabianova@vzbb.sk; 23 Epidemiology Health Department, Regional Authority of Public Health, 97556 Banská Bystrica, Slovakia; beresova@vzbb.sk; 24 Health Promotion Department, Regional Authority of Public Health, 97556 Banská Bystrica, Slovakia; adamcakova@vzbb.sk; 25 Health Informatics Department, Regional Authority of Public Health, 97556 Banská Bystrica, Slovakia; roman.nedela@gmail.com; 26 Infectology Clinic, Central Military Hospital, 03426 Ružomberok, Slovakia; anna.lesnakova@ku.sk; 27 Occupational Medicine Clinic, University Hospital, 83105 Bratislava, Slovakia; holcikova.jana@gmail.com; 28 Department of Medical and Surgical Sciences, University of Bologna, 40138 Bologna, Italy; paolo.boffetta@unibo.it (P.B.); mahsa.abedini@unibo.it (M.A.); giorgia.ditano2@unibo.it (G.D.); shuffield.asafo2@unibo.it (S.S.A.); giovanni.visci@studio.unibo.it (G.V.); francesco.violante@unibo.it (F.S.V.); carlotta.zunarelli@studio.unibo.it (C.Z.); 29 Stony Brook Cancer Center, Stony Brook University, Stony Brook, NY 11794, USA; 30 IRCCS, Azienda Ospedaliero Universitaria di Bologna, 40138 Bologna, Italy; 31 Unit of Epidemiology and Medical Statistics, Department of Diagnostics and Public Health, University of Verona, 37134 Verona, Italy; giuseppe.verlato@univr.it

**Keywords:** breakthrough infections, health workers, COVID-19, occupational and socio-demographic determinants, SARS-CoV-2 vaccination

## Abstract

Background: The research aimed to investigate the incidence of SARS-CoV-2 breakthrough infections and their determinants in a large European cohort of more than 60,000 health workers. Methods: A multicentric retrospective cohort study, involving 12 European centers, was carried out within the ORCHESTRA project, collecting data up to 18 November 2021 on fully vaccinated health workers. The cumulative incidence of SARS-CoV-2 breakthrough infections was investigated with its association with occupational and social–demographic characteristics (age, sex, job title, previous SARS-CoV-2 infection, antibody titer levels, and time from the vaccination course completion). Results: Among 64,172 health workers from 12 European health centers, 797 breakthrough infections were observed (cumulative incidence of 1.2%). The primary analysis using individual data on 8 out of 12 centers showed that age and previous infection significantly modified breakthrough infection rates. In the meta-analysis of aggregated data from all centers, previous SARS-CoV-2 infection and the standardized antibody titer were inversely related to the risk of breakthrough infection (*p* = 0.008 and *p* = 0.007, respectively). Conclusion: The inverse correlation of antibody titer with the risk of breakthrough infection supports the evidence that vaccination plays a primary role in infection prevention, especially in health workers. Cellular immunity, previous clinical conditions, and vaccination timing should be further investigated.

## 1. Introduction

Since its first identification [1], Severe Acute Respiratory Syndrome Coronavirus (SARS-CoV-2) infections have caused about 15 million deaths attributable to Coronavirus disease (COVID-19) worldwide [2]. The rapid development of multiple successful vaccines strongly impacted the clinical burden, preventing the evolution of the severe symptomatic disease and, consequently, mortality [3,4,5].

Vaccines also reduced the transmission rates of SARS-CoV-2, particularly in the first 4–6 months after the vaccination, due to a more rapid decline in viral load and decreased viability of the virus shed by vaccinated individuals and being less likely to be culture-positive [6].

However, the continuous emergence of new viral variants with different characteristics perpetuates viral transmission and threatens the vaccine’s efficacy. So far, five variants responsible for new resurgence waves have been isolated [7]. The Omicron variant is the most recent designated variant of concern (VOC) due to its remarkable ability to escape vaccine and infection-induced immunity and its resistance to therapeutic antibodies [8].

However, as early a few months after the SARS-CoV-2 outbreak, a critical issue emerged related to the fact that the virus continuously evolves, and changes in the genetic code (caused by genetic mutations or viral recombination) occur during genome replication, so the concept of SARS-CoV-2 variants was introduced [7,8].

Therefore, since late 2020, the appearance of variants that posed an increased risk to global public health prompted the characterization of specific Variants of Interest (VOIs) and Variants of Concern (VOCs) to prioritize global monitoring and research and ultimately inform the ongoing response to the COVID-19 pandemic [9].

To date, five variants responsible for new resurgence waves have been isolated: Alpha B.1.1.7; Beta B.1.351; Gamma P.1; Delta B.1.617.2; and the Omicron variant B.1.1.529, the most recent designated variant of concern (VOC) due to its remarkable ability to escape vaccine and infection-induced immunity and its resistance to therapeutic antibodies [10,11].

The incidence of breakthrough infections (BI), defined as infections occurring in fully vaccinated people, has been increasingly reported, even though it is associated with asymptomatic and mild diseases [12,13,14]. Reasons for the occurrence of BI include a combination of different factors, such as an ageing population, high-risk occupation, level of population immunity, partial viral escape, drop-in antibody activity over time, and uncertain duration of immune memory.

Early reports provided evidence of the decline in antibody titers a few months after completing the two-dose COVID-19 vaccine regimen [15,16,17]. Preliminary laboratory findings on the distribution of S-antibody levels at subsequent time points after vaccination showed a decrease for both BNT162b2 (Pfizer–BioNTech) and ChAdOx1 nCoV-19 (Oxford–AstraZeneca), a trend confirmed for different subgroup stratification [18]. Likewise, neutralizing antibody titers following SARS-CoV-2 infection drop over time [19]. A study involving all fully vaccinated Israeli adult population documented an increased infection rate with increasing time since vaccination, with a 1.6-to-2.1-fold higher rate of infection associated with a two-month increase in the time difference [20]. The growing evidence of the waning of vaccine-induced protection and natural immunity after 3–6 months has reinforced the need for a third dose, which has been shown to restore levels of neutralizing antibodies [21]. An observational study compared health workers (HWs) who received the two-dose regimen at least six months earlier with HWs who received a booster dose. A significantly lower BI rate in boosted individuals showed strong protection against SARS-CoV-2 infection conferred by the third dose [22]. However, the determinants of vaccine response are still unclear. So far, no striking variables (such as age, sex, or comorbidities) were identified as significantly associated with higher IgG titers [16].

Notably, the third dose increases neutralizing titers against VOCs, including the Omicron variant, even at a lower level [23,24,25].

Less is known about the B cell response in vaccinated individuals, but some studies have shown that vaccines induce the production of memory B cells, whose increase remains durable even at 6–9 months post-vaccination [26].

Evaluating the incidence of BI and the long-term efficacy of vaccines is crucial to drive public health decisions regarding timing of vaccination, screening, health surveillance, restriction measures, and access to the workplace [27].

In most European countries, HWs undergo constant surveillance due to their risk of exposure to SARS-CoV2 and relevance for Public Health; therefore, they represent an important population to evaluate current strategies’ effectiveness and delineate future interventions.

The ORCHESTRA project is a three-year international research project, building up different European cohorts by applying harmonized protocols for data collection, data sharing, sampling, and follow-up. The Work Package 5 of ORCHESTRA focuses on HWs to define the incidence and prevalence of SARS-CoV-2 infection, the duration of immunity induced by natural infection and vaccination by collecting biological samples at fixed time-points, and risk of reinfection and breakthrough infections.

To date, a number of single-center studies investigating the role of significant determinants on SARS-CoV-2 infections in HWs, from medium-sized samples, have inconsistent results on the possible role of age, sex, and job title [28].

This research aimed to investigate the incidence of SARS-CoV-2 breakthrough infections and their determinants in a European HWs cohort.

## 2. Materials and Methods

### 2.1. Design and Setting

A multicenter retrospective cohort study of HWs (including workers dedicated to clinical activities, as well as technicians and administrative workers) from 12 European centers involved in the ORCHESTRA project was performed. Data were collected from the following healthcare settings, mainly University Hospital places: Italy (Bari, Bologna, Brescia, Modena, Padova Perugia, Torino, Trieste, and Verona), Romania, Slovakia, and Spain (Oviedo). This study followed the Strengthening the Reporting of Observational Studies in Epidemiology (STROBE) reporting guidelines [29].

### 2.2. Vaccination, Case Definition, and Inclusion Criteria

HWs enrolled in a continuous follow-up since the beginning of pandemic were included. The analysis was conducted on HWs that completed the vaccination course (fully vaccinated), according to the specified vaccine timing, since the 14th day after fully vaccination from February 2021 to November 2021. HWs not fully vaccinated, and those infected after the third dose, were excluded.

A total of 96.26% (N = 61,771) of enrolled HWs had received BNT162b2 (Pfizer–BioNTech), while 3.39% (N = 2176) recieved mRNA-1273 (Moderna), 0.28% (N = 182) ChAdOx1 nCoV-19 (Oxford–AstraZeneca), and 0.07% (N = 43) Ad26.COV2.S (Johnson).

Vaccine BI was defined as PCR-confirmed COVID-19 ≥14 days following the course completion of the four vaccine doses [30].

### 2.3. Outcome and Data Collection

The incidence of SARS-CoV-2 BI and their characteristics (sex, age, job-title, previous infections, SARS-CoV-2 antibody titer) were investigated.

Clinical and laboratory data were collected using a standardized data collection form.

SARS-CoV-2 infection was diagnosed by positive real-time reverse-transcriptase polymerase chain reaction (RT-PCR), performed using various commercially available assays in different clinical laboratories. Each center adopted a different and variable timing for screening programs through nasopharyngeal swabs, according to epidemiological contexts and local regulations.

S-specific IgG antibodies against SARS-CoV-2 titration were measured within 21 days and three months after the first vaccine dose. In order to compare the results provided by the laboratories of the included centers, a normalization approach was applied. Antibody levels were log-transformed to take into account the skewness of the distribution, and log-transformed results were normalized by dividing them by the center-specific standard error.

### 2.4. Statistical Analysis

The primary statistical analysis utilized individual data from a subgroup of centers and consisted of survival analysis, where BI was the terminal event, and time elapsed since the second dose was the time variable. A univariable survival analysis was accomplished by Kaplan–Meier survival curves and a log-rank test and a multivariable survival analysis by centre-stratified Cox regression models, where sex, age, job-title, and infection pre-vaccination against SARS-CoV-2 were the explanatory variables. Proportional hazard assumptions of the Cox model were tested based on Schoenfeld residuals. In addition, the proportionality assumption was checked by graphic methods: it was verified whether -ln[-ln[survival]] curves for each category of risk factors were parallel when plotted vs ln [analysis time]. Moreover, a multinomial regression model compared the risk of SARS-CoV-2 infection before and after vaccination. The response variable was 0 = no infection/1 = infection before vaccination/2 = infection after vaccination; predictors were sex, age, and job title. Results were synthesized through the relative risk ratios (RRRs), adjusting standard errors for intra-center correlation. The significance of the association between timing of vaccination course completion and cumulative incidence of breakthrough infection in consecutive periods was evaluated by a chi-square test.

A secondary statistical analysis was performed on aggregate data from all of the 12 participating centers and was accomplished by meta-analyses. A test of heterogeneity was applied to evaluate variability among studies. The I-squared statistic was computed, which indicates the proportion of total variation among the effect estimates of different studies attributed to heterogeneity rather than sampling error. When the heterogeneity test was not significant (*p* > 0.050) and I-squared 2 was less than 30% [31,32], heterogeneity was ruled out. In this case, a fixed-effects model was adopted for the evaluation of the results, which were pooled using the method of Mantel and Haenszel. Otherwise, a random-effects model was used, and the pooling of results was done using the DerSimonian and Laird method [33]. The level of statistical significance was set at 5%, and confidence intervals (CI) were calculated at 95%. The results were displayed graphically using forest plots. Stata^®^ software 15 (Stata Corp LP, College Station, TX, USA) was used for statistical analysis.

### 2.5. Ethical Approval

The research was performed following the 1964 Declaration of Helsinki standards and its later amendments. The study was approved (No.436 14 October 2021) by the Italian Medicine Agency (AIFA) and the Ethics Committee of the Italian National Institute of Infectious Diseases (INMI) Lazzaro Spallanzani. All the Cohorts collected loco-regional ethical approvals.

## 3. Results

During the study period, 64,172 HWs were enrolled, mainly provided by the Italian cohorts (75%). Most HWs were female (70.5 %), and almost half were over 40 years. Nurses (35.9%) and physicians (29.6%) were the most represented job title. A total of 797 BIs were detected, corresponding to an overall cumulative incidence of 1.2% (Table 1).

### 3.1. Primary Analysis on Individual Data

The primary analysis was conducted using data of 31,837 HWs belonging to eight centers.

The temporal trends of BI are shown in Supplementary Figure S1. The trend was similar in Italian centers, with a slightly higher cumulative incidence in Modena and a lower incidence in Bari. Cumulative incidence rose in the first 12 weeks after the full vaccination course, remained stable in the subsequent 12 weeks, and rose again thereafter. In Romania and Slovakia, the late increase was not observed.

Results of the main risk factors of BI from the Cox-regression analysis are displayed in Table 2. Age and previous infection were the only two significant determinants (*p* < 0.001). The risk of BI decreased by about 20% for an additional decade of age and was 4–5-times lower in people with pre-vaccination SARS-CoV-2 infection.

**Figure 1:**
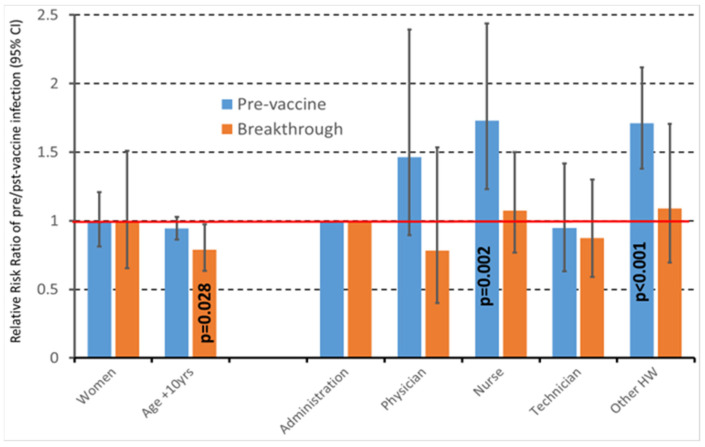
Impact of socio-demographic characteristics on the risk of either pre-vaccine infection (blue columns) or breakthrough infection (orange columns) in the eight centers (31,837 HWs) providing individual data. Columns are Relative Risk Ratios (RRR), and bars are 95% confidence intervals. RRRs were estimated by multinomial logistic regression, adjusting standard errors for intra-center correlation.

Figure 1 presents changes in the risk profile for SARS-CoV-2 infections before and after a full vaccination course. It can be appreciated that the association of age and job title with SARS-CoV-2 infection completely changed before and after vaccination. In detail, nurses and other HWs were at a higher risk of SARS-CoV-2 infection than administrative employees before the full vaccination course but not thereafter. Conversely, age was not associated with SARS-CoV-2 infection before vaccination, while the risk of SARS-CoV-2 infection was inversely related to age thereafter.

The BI cumulative incidence in three consecutive periods (May/June, July/August, September/October 2021) was calculated in 24,358 HWs from six Italian centers. The median time lag between second dose administration and BI was 136.5 days (p25–p75 = 60–201 days). Considering three different time frames, the observed cumulative incidence of BIs for HWs completing the vaccination course during January, February, and March ranged between 19 and 50, 12 and 31, and 12 and 63 per 10,000 HWs, respectively (*p* = 0.163) (Supplementary Figure S2).

### 3.2. Secondary Analyses on Aggregate Data

**Figure 2:**
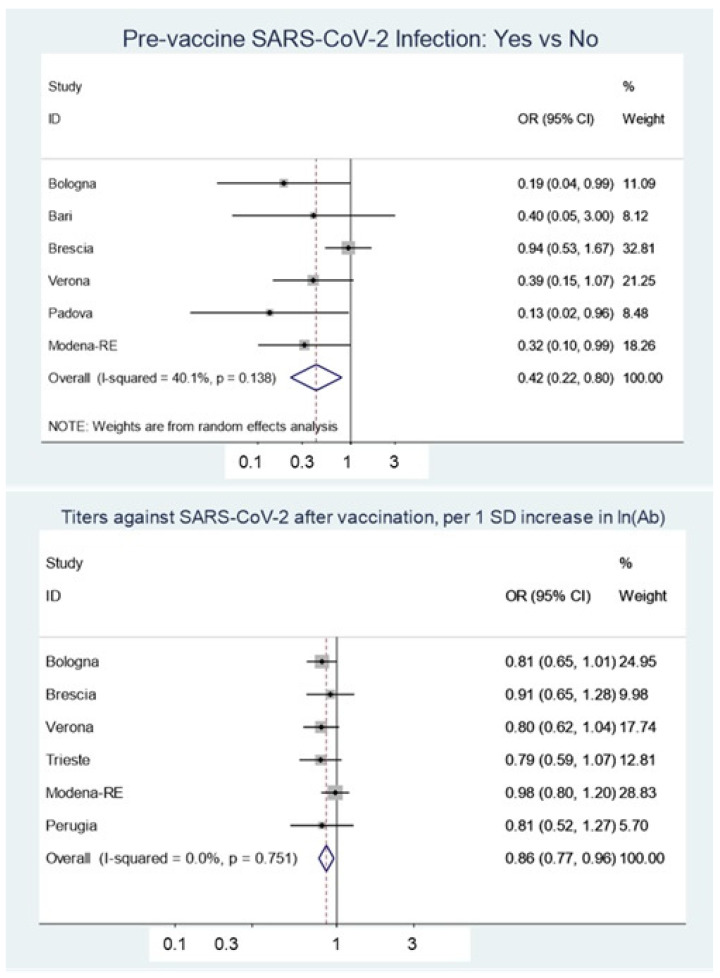
Forest plots evaluating the effect of SARS-CoV-2 infection before vaccination (upper panel) and antibody titers against SARS-CoV-2 after vaccination (lower panel) on the risk of breakthrough infection. A random-effect model was used for the former and a fixed-effects model for the latter. Odds ratio (OR) estimates for single centers are shown in boxes, and the pooled estimate is shown as a diamond. Error bars and values in parentheses indicate 95% confidence intervals.

Table 3 shows the meta-analysis results on aggregate data from all 12 centers. Sex, age, and job title had no statistically significant effect, while previous SARS-CoV-2 infection and standardized antibody titer were inversely related to the risk of breakthrough infection (Table 3; Figure 2).

## 4. Discussion

In the HWs ORCHESTRA cohorts, the cumulative incidence of BIs, from February 2021 to November 2021, was 1.2%, with differences between centers that could be explained by different epidemiological infection patterns in distinct regions. In addition, each center had its timing for nasopharyngeal screening, based on epidemiological contexts and local regulations. Consequently, it was likely that centers adopting a screening approach with frequent testing and fast turnaround detected a higher number of asymptomatic infections, in line with what has been reported in the literature [34].

We aimed to investigate the relationship between the occurrence of BIs among HWs and its association with occupational and socio-demographic characteristics and to provide a comparison with their role in pre-vaccine infections.

In the pre-vaccination phase, the risk of SARS-CoV-2 infection was not affected by either sex or age, whereas risk differences were detected within job categories. In detail, using administrative health personnel as a reference, nurses and other HWs were at greater risk, with statistically significant differences, as were doctors, although without statistical significance. Our results agree with the study of Al Youha S et al. [35], conducted during the second wave in the pre-vaccine era, showing that among HWs, the odds of contracting SARS-CoV-2 infection were the highest among nurses (adjusted OR 1.77, 95% CI 1.15−2.71) with doctors as a reference. No significant differences were found within sex. As reported in the meta-analysis by Gomez-Ochoa SA et al. [36] the high number of positive nurses for SARS-CoV-2 could be explained by their major involvement in patient care.

The comparison with the literature must also consider the pandemic trend and the different waves that occurred worldwide. Regarding SARS-CoV-2 incidence during the first wave, in a previous Italian monocentric study, partially overlapped with the present population, Porru et al. [37] reported that the risk of SARS-CoV-2 detection was not significantly affected by sex, age, or type of job while, from an analysis in the same period in an HWs cohort from six Italian centers, partially overlapping with the present population, Boffetta et al. [38] reported no differences in the risk of infection according to job title and age, while females were at a lower risk of infection than males. According to this evidence, in a rapid review updating previous papers, Chou R et al. [39] reported no association between sex or job title and risk for SARS-CoV-2 infection.

In the present study, the determinants of SARS-CoV-2 infection in the post-vaccinal phase were analyzed on both individual (eight centers) and aggregated (12 centers) data.

The analysis of individual data showed that age (per 10-year increase) and previous infection were inversely associated with the incidence of BI. In contrast, in an aggregated data analysis, age had no significant statistical effect. Previous SARS-CoV-2 infection and standardized antibody titer were inversely related to the risk of breakthrough infection.

The currently available literature provides conflicting views on the role of occupational and socio-demographic characteristics. Some studies have shown an association between age and increased risk, while others have not found any association [28]. It should be noted that the age effect could be appraised through other determinants, including comorbidities and the infection severity. Increased social contacts and assignment might influence increased risk in younger people to higher risk wards [40]. In addition, a healthy worker effect could also occur in a population of HWs.

Regarding sex and job title, our study is in line with the body of the available literature. There is no association between risk for SARS-CoV-2 infection in HWs and sex or job-title. Concerning the Job-title, the difference between pre-vaccination and breakthrough risk of infection may be due to the change in the type of exposure during the different waves of the pandemic, with a preponderance of household or private setting exposure compared to occupational exposure [28]. Moreover, during the first pandemic wave, global shortages of masks, respirators, face shields, and gowns, caused by surging demand and supply chain disruptions, led to efforts to conserve personal protective equipment through extended use or reuse, with a subsequent increased risk of COVID-19 [41]. The later implementation of safety procedures and the increased availability of protective equipment may have influenced the evolution of the risk scenario.

In this research, SARS-CoV-2 titration was a protective factor for breakthrough infection.

According to available literature, there is a persistence of protection lasting at least six months after the second dose, despite a waning of antibody titer from the vaccine second dose, for both BNT162b2 and ChAdOx1 nCoV-19 [15,20].

Furthermore, in our sample, with a follow-up 7–9 months after the full vaccination, we did not observe a significant variation in the incidence of breakthroughs for HWs vaccinated at different times, while Mizrahi et al. [18] did.

A SARS-CoV-2 previous infection elicits a protective antibody response that is reinforced by vaccination, in line with other research available. Wei et al. found that antibody levels among previously infected individuals were higher than those in non-previously infected individuals. The boosting effect of the second dose was less as titers were very high already after the first dose. Hall et al. [42] also showed that two doses of the BNT162b2 vaccine were associated with increased short-term protection against SARS-CoV-2 infection; this protection waned considerably after six months, and infection-acquired immunity boosted with vaccination remained high more than one year after infection.

### Strengths and Limitations

Major strengths of the paper are the sample size, the largest (to the best of our knowledge) in the available published literature on HWs; the multicentric recruitment; and the significant contribution from countries of the Mediterranean area, Italy and Spain, and Romania and Slovakia, strongly affected by the pandemics.

These aspects lend power to the study and provide good stability of results and generalizability. This is coupled with a solid research methodology, also supported by the laboratory data from certified laboratory centers. In addition, the normalization made it possible to make the data comparable despite the different titration techniques.

In addition, the time frame of observation was wide and enabled the evaluation of the entire time available before the third dose.

It is also an ongoing study that will be followed by the papers on the BI after the third dose.

The study also has some limitations to be disclosed. The analysis of individual data was only conducted on about half of the population (30,000 HWs), albeit a large one, for which individual data were available. Furthermore, due to the unavailability of data, it was impossible so far to assess the BIs’ clinical characteristics, to distinguish between symptomatic and asymptomatic cases and the source case, and to distinguish between occupational and household exposure. Information on clinical determinants, such as previous clinical conditions, of HWs was also missing. These will be addressed in the ongoing follow-up via ad hoc online questionnaires.

## 5. Conclusions

This study confirmed that SARS-CoV-2 infections occur even after a full vaccination course. The risk of infection is not influenced by age, sex, and job title, while a higher SARS-CoV-2 antibody titer and a previous positive molecular test result in certain protections. Interestingly, regarding job title, its effect is variable between pre- and post-vaccine administration, with the effect rising in post. This finding will be further investigated together with the trend of BI after the third dose.

## Figures and Tables

**Table 1:** Breakthrough infections, demographic, and occupational characteristics of 64,172 Health Workers from 12 European centers.

		Positive	Sex (%)		Job Title (%)					Age (10 Years %)			
Centre	N	Cases (%)	Male	Female	Administr.	Technician	Nurse	Physician	Other HW	<30	30–39	40–49	≥50
**Individual data available**													
Verona	6404	97 (1.5)	1979 (30.9)	4425 (69.1)	514 (8.0)	579 (9.0)	2176 (34.0)	2167 (33.8)	968 (15.1)	1068 (16.7)	1635 (25.5)	1241 (19.4)	2460 (38.4)
Padua	6208	92 (1.5)	1901 (30.6)	4307 (69.4)	542 (8.8)	428 (7.0)	2370 (38.5)	1844 (30.0)	973 (15.8)	850 (13.7)	1214 (19.6)	1400 (22.6)	2744 (44.2)
Trieste	3559	59 (1.7)	1013 (31.8)	2169 (68.2)	166 (5.2)	144 (4.5)	1313 (41.2)	527 (16.5)	1038 (32.6)	220 (6.9)	554 (17.4)	854 (26.8)	1554 (48.8)
Modena	5250	90 (1.7)	1550 (29.5)	3699 (70.5)	268 (5.2)	179 (3.5)	1846 (36.0)	1603 (31.2)	1239 (24.1)	946 (18.0)	1409 (26.8)	1140 (21.7)	1755 (33.4)
Perugia	2364	30 (1.3)	789 (33.4)	1575 (66.6)	170 (7.2)	327 (13.8)	1002 (42.4)	514 (21.7)	351 (14.9)	31 (1.3)	468 (19.8)	543 (23.0)	1322 (55.9)
Bari	5923	38 (0.6)	2330 (39.3)	3593 (60.1)	379 (6.4)	200 (3.4)	1612 (27.2)	2884 (48.7)	848 (14.3)	901 (15.2)	1356 (22.9)	1081 (18.3)	2585 (43.6)
Slovakia	671	9 (1.3)	106 (15.8)	565 (84.2)	74 (11.1)	36 (5.4)	227 (34.0)	83 (12.4)	247 (37.0)	75 (11.2)	96 (14.3)	220 (32.8)	280 (41.7)
Romania	1458	11 (0.8)	276 (18.9)	1182 (81.1)	69 (4.7)	13 (0.9)	172 (11.8)	1080 (74.1)	124 (8.5)	89 (6.1)	179 (12.3)	440 (30.2)	750 (51.4)
**Subtotal**	31,837	426 (1.3)	9944 (31.6)	21,515 (68.4)	2182 (7.0)	1906 (6.1)	10,718 (34.3)	10,702 (34.2)	5788 (18.5)	4180 (13.3)	6911 (22.0)	6919 (22.0)	13,450 (42.8)
**Aggregated data only**													
Turin	8787	75 (0.9)	2427 (27.6)	6360 (72.4)	1225 (13.9)	1169 (13.3)	3219 (36.6)	1841 (20.9)	1333 (15.2)	1063 (12.1)	1197 (13.6)	2033 (23.1)	4491 (51.1)
Brescia	8903	134 (1.5)	2446 (27.5)	6457 (72.5)	985 (11.1)	702 (7.9)	2855 (32.1)	2642 (29.7)	1719 (19.3)	1386 (15.6)	1968 (22.1)	2015 (22.6)	3534 (39.7)
Bologna	7229	95 (1.3)	2417 (33.4)	4812 (66.6)	274 (3.9)	705 (10.0)	2474 (34.9)	1998 (28.2)	1631 (23.0)	1395 (19.3)	1934 (26.7)	1474 (20.4)	2426 (33.6)
Oviedo	7416	67 (0.9)	1569 (21.2)	5847 (78.8)	691 (9.3)	413 (5.6)	3494 (47.1)	1615 (21.8)	1203 (16.2)	582 (7.8)	1290 (17.4)	1760 (23.7)	3784 (51.1)
**Total**	64,172	797 (1.2)	18,803 (29.5)	44,991 (70.5)	5357 (8.4)	4895 (7.7)	22,760 (35.9)	18,798 (29.6)	11674 (18.4)	8606 (13.5)	13,300 (20.8)	14,201 (22.3)	27,685 (43.4)

**Table 2:** Main risk factors of breakthrough infection in the eight centers (31,837 HWs). Hazard ratios and *p*-values were obtained by a Cox regression model, including sex, age, job title, and pre-vaccine SARS-CoV-2 infection, stratifying by center.

	Hazard Ratio (95% CI)	*p*-Value
Sex (Women vs. men)	0.98 (0.79–1.23)	0.899
**Age (increase by 10 years)**	**0.81 (0.74–0.88)**	**<0.001**
Job title		
Administrative	1 (reference)	
Physician	0.82 (0.53–1.27)	0.374
Nurse	0.98 (0.64–1.49)	0.920
Technician	0.75 (0.41–1.36)	0.341
Other HW	1.03 (0.66–1.61)	0.910
**Previous infection (Yes vs. No)**	**0.23 (0.12–0.45)**	**<0.001**

Significant results are highlighted in bold. HW = Health Worker.

**Table 3:** Summary of meta-analyses performed on all 12 centers with available aggregate data.

	Centres	Pooled OR (95% CI)	*p*-Value	I-Squared
**Gender: women vs. men**	11	0.91 (0.70–1.19)	0.488	**51.6% (*p* = 0.024)**
**Age (increased by 10 years)**	12	0.91 (0.80–1.05)	0.188	**45.5% (*p* = 0.043)**
Job title (ref. administration)				
**Physician**	11	1.05 (0.62–1.79)	0.858	**51.6% (*p* = 0.024)**
Nurse	12	1.29 (0.93–1.80)	0.130	0.0% (*p* = 0.562)
Technician	10	1.37 (0.86–2.18)	0.191	0.0% (*p* = 0.777)
Other HW	11	1.20 (0.82–1.74)	0.353	0.0% (*p* = 0.516)
**Previous COVID-19**	6	**0.425 (0.225–0.80)**	**0.008**	**40.1%** (*p* = 0.138)
**Ln (Antibody titre) (+1 sd)**	6	**0.86 (0.775–0.96)**	**0.007**	0% (*p* = 0.751)

Significant results are highlighted in bold.

## Data Availability

The datasets generated during the current study are not publicly available, because they contain sensitive data to be treated under data protection laws and regulations. Appropriate forms of data sharing can be arranged after a reasonable request to the first author.

## Supplementary Materials

The following supporting information can be downloaded at: https://www.mdpi.com/article/10.3390/vaccines10081193/s1. Figure S1: Cumulative incidence of breakthrough infection in the eight centers providing individual data, estimated by the Kaplan–Meier method; Figure S2: Cumulative incidence of breakthrough infections in three different consecutive periods (May/June, July/August, September/October 2021).
