# Supplementary figures and images for: SARS-CoV-2 Breakthrough Infections: Incidence and Risk Factors in a Large European Multicentric Cohort of Health Workers
Source: Vaccines (Basel). 2022 Jul 27;10(8):1193. doi: 10.3390/vaccines10081193 (PMC9415790; doi:10.3390/vaccines10081193)

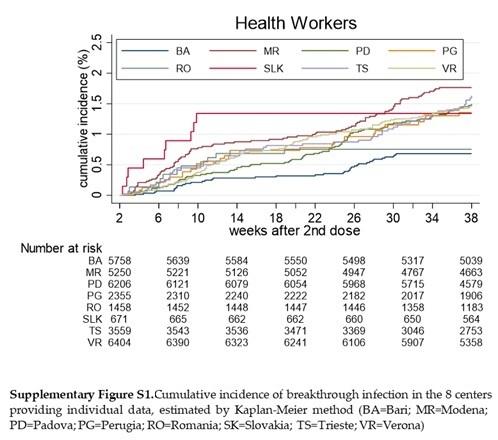

Supplement: Supplementary file 1 [file vaccines-10-01193-s001.zip › Figure S1.jpg]

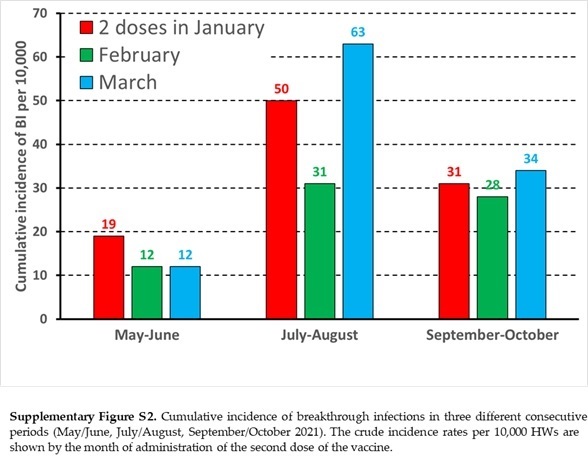

Supplement: Supplementary file 1 [file vaccines-10-01193-s001.zip › Figure S2.jpg]
